# Selection of reference genes for gene expression studies in human bladder cancer using SYBR-Green quantitative polymerase chain reaction

**DOI:** 10.3892/ol.2022.13205

**Published:** 2022-01-18

**Authors:** Chuanxia Zhang, Yong Qiang Wang, Guangyi Jin, Song Wu, Jun Cui, Rong-Fu Wang

Oncol Lett 14: 6001-6011, 2017; DOI: 10.3892/ol.2017.7002

Following the publication of the above article, the authors have realized that they inadvertently included the same data for the HSP90AB1 and ATP5B experiments shown in Fig. 1A on p. 6005. This error arose during the compilation of the figure; after having re-examined their original data, the authors have realized that the data were chosen incorrectly for the ATP5B experiment.

The revised version of Fig. 1, including all the correctly assembled data for Fig. 1A, is shown on the next page. Note that the error made during the assembly of this figure did not affect the overall conclusions reported in the paper. All the authors agree with the publication of this corrigendum, with the exception of Rong-Fu Wang, who is no longer contactable. The authors are grateful to the Editor of *Oncology Letters* for allowing them the opportunity to publish this, and also apologize to the readership for any inconvenience caused.

## Figures and Tables

**Figure 1.**
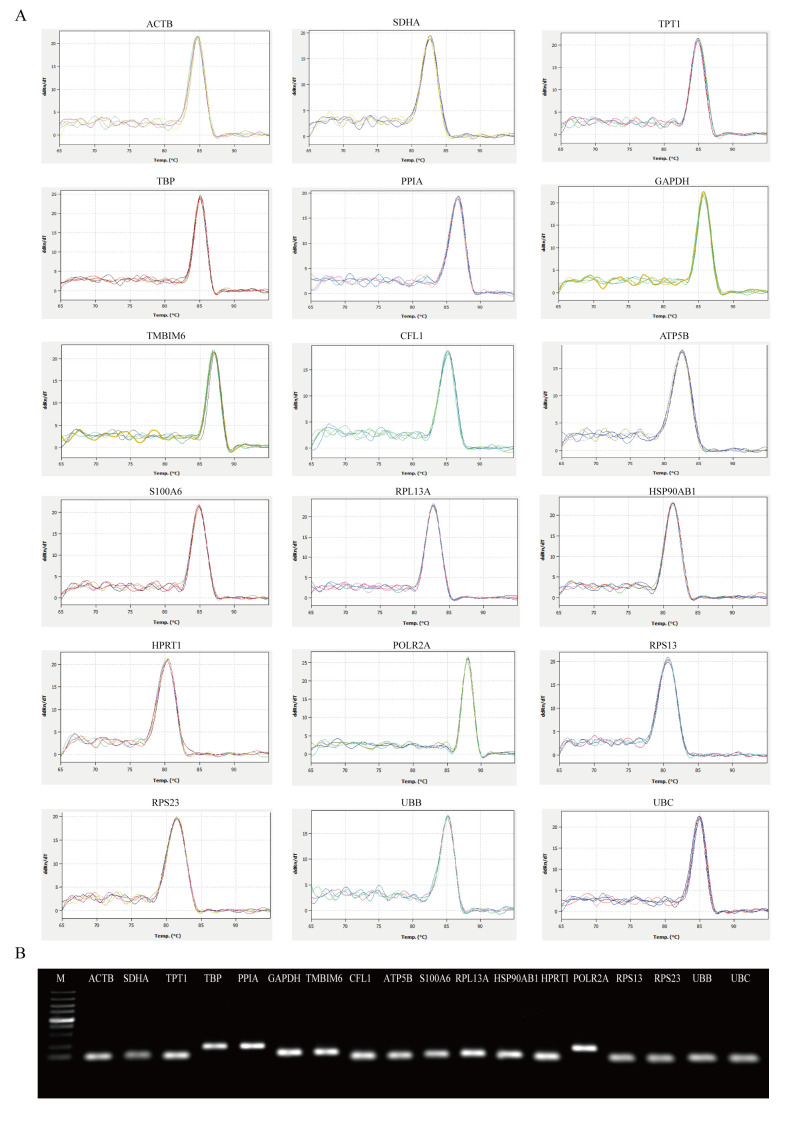
Primer specificity analysis for the 18 candidate reference genes. (A) Melting curves of the 18 reference genes exhibiting single peaks. (B) Agarose gel electrophoresis (1.2%) exhibited a single and specific polymerase chain reaction product of each reference gene. M, marker lane (from bottom to top: 100, 250, 500, 750, 1,000, 1,500, 2,000, 3,000 and 5,000 bp). See Table II for gene name definitions.

